# Commentary: Antidepressant Use During Acute Inpatient Care is Associated With an Increased Risk of Psychiatric Rehospitalisation Over a 12-Month Follow-Up after Discharge

**DOI:** 10.3389/fpsyt.2019.00990

**Published:** 2020-02-05

**Authors:** Erich Seifritz

**Affiliations:** Department of Psychiatry, Psychotherapy and Psychosomatics, Psychiatric Hospital, University of Zurich, Zurich, Switzerland

**Keywords:** depression, antidepressant, prognosis, naturalistic study, nearest neighbor propensity score

The research report by Hengartner et al. (1) in this journal claims that “antidepressant use during acute inpatient care is associated with an increased risk of psychiatric rehospitalisation over a 12-month follow-up after discharge”. The authors employed a matched pairs comparison of inpatients who were referred with a mixed range of diagnoses to a psychiatric hospital. Patients were matched *via* nearest neighbor propensity scores (2) and divided into two groups of N = 45 with respect to whether they had been prescribed an antidepressant or not, i.e. whether they were either antidepressant users or non-users. The matching procedure was based on 14 covariates that assessed sociodemographic items, psychosocial impairments, functioning deficits, and illness severity.

While this general methodological approach in naturalistic clinical studies is widely accepted (3), the conclusions made in this particular paper are not warranted. Here, the covariates employed for the matching procedure are, unfortunately, incomplete with regard to the intervention of interest, namely the prescription or non-prescription of antidepressant medication. In fact, none of the 14 matching parameters addressed presence or absence of any reason(s) for clinically meaningful in- or off-label antidepressant use (4, 5), which would be mandatory for the research question under study. Most importantly, assuming a non-random prescription of antidepressants, the patients in the two groups must have been fundamentally different with respect to relevant clinical parameters (6, 7) which, in turn, would be likely to predict outcome as reflected in rehospitalization rate (8). Obviously, these relevant critical factors were not assessed in the analysis. Incidentally, rehospitilization rate is not actually considered to be a marker of treatment quality and is thus a somewhat questionable outcome measure (9).

The paper’s conclusion is thus the ramification of a hidden selection bias due to omission of information of fundamental relevance. To illustrate the pitfalls of this faulty approach, a similar erroneous conclusion would be, for example, that cytostatic drugs are harmful because subjects who use cyotostatic drugs are more likely to finally die from cancer than subjects not using such drugs, even when the two groups had been matched perfectly for age, gender, and a series of more or less useful health parameters. While this association in itself is numerically true, it is logically wrong to imply the causal relationship that cytostatic drugs produce cancer.

In their paper, Hengartner et al. (1) include an extensive section on study limitations, in which they reason that the methodological flaws do not restrict the main conclusion, as underscored in the paper’s title. However, because the results and main messages are based on a fundamental logical error, and because omitting important covariates in propensity matching is a detrimental failure (10), the key message is not justified by the methodology used. Therefore, the paper is certainly misleading and, furthermore, potentially harmful.

## Author Contributions

ES has drafted and written the commentary, with support by experts in methodology.

## Funding

Funding by University of Zürich.

## Conflict of Interest

The author declares that the research was conducted in the absence of any commercial or financial relationships that could be construed as a potential conflict of interest.
